# Chronic treatment with a metabotropic glutamate receptor 2 camelid nanobody accelerates amyloidogenesis in Alzheimer’s disease

**DOI:** 10.1002/alz.088294

**Published:** 2025-01-09

**Authors:** Pierre‐André Lafon, Jianfeng Liu, Veronique Perrier, Philippe RONDARD

**Affiliations:** ^1^ University of Montpellier, Montpellier, Occitanie France; ^2^ University of Montpellier, Montpellier France

## Abstract

**Background:**

Immunotherapy of Alzheimer’s disease (AD) is a promising approach to reducing the accumulation of beta‐amyloid, a critical event in the onset of the disease. Targeting the group II metabotropic glutamate receptors, mGluR2 and mGluR3, could be important in controlling Aβ production, although their respective contribution remains unclear due to the lack of selective tools.

**Method:**

5xFAD mice were chronically treated by a brain penetrant camelid single domain antibody (VHH or nanobody) that is an activator of mGluR2. Abeta amyloids after this treatment were measured by FRET‐based technologies (HTRF). HTRF techniques were also developed to measure the direct interaction between APP and mGluR2 or mGluR3 at the surface of transfected cells. Other resonance energy transfer techniques were also used to measure the influence of the nanobody on mGluR2 and mGluR3 internalization.

**Result:**

Enhancing mGluR2 activity increases Aβ_1‐42_ peptide production whereas activation of mGluR3 has no effect. We show that such a difference likely results from the directly interaction of APP with mGluR3, but not with mGluR2, that prevents APP degradation to Aβ_1‐42_ peptides. We then show that chronic treatment with a brain‐penetrating mGlu2 potentiating nanobody of the AD model 5xFAD mice accelerated amyloid aggregation and exacerbated memory deficits, but had no effect in control mice.

**Conclusion:**

A selective mGluR2 activation exacerbates AD disease development suggesting that therapeutic benefices could be obtained with blockers of this receptor.

